# Comparison of Supine and Lateral Decubitus Positions for Total Hip Arthroplasty with the Direct Lateral Approach in Overweight and Obese Patients

**DOI:** 10.1155/2020/8684067

**Published:** 2020-02-18

**Authors:** Olcay Güler, Sidar Öztürk, Ferit Tufan Özgezmez, Mehmet Halis Çerçi

**Affiliations:** ^1^Department of Orthopedics and Traumatology, Istinye University, Medical Faculty, Istanbul, Turkey; ^2^Department of Orthopedics and Traumatology, Derindere Hospital, Istanbul, Turkey; ^3^Department of Orthopedics and Traumatology, Adnan Menderes University, Medical Faculty, Aydın, Turkey; ^4^Department of Orthopedics and Traumatology, Nisa Hospital, Istanbul, Turkey

## Abstract

**Background:**

The purpose of our study is to compare the results of supine and lateral decubitus positions for total hip arthroplasty (THA) with the direct lateral (DL) approach in overweight and obese patients.

**Methods:**

Patients who had a THA with the DL approach using the lateral decubitus position (LD group) (*n* = 54) or supine position (S group) (*n* = 54) or supine position (S group) (

**Results:**

Both groups did not differ from each other by means of age, gender, BMI, and affected side (*p* = 0.814, *p* = 0.814, *p* = 0.814, *p* = 0.814, *p* = 0.814, *p* = 0.814, *p* = 0.814, *p* = 0.814,

**Conclusions:**

The LD and S groups had comparable functional outcomes one year postoperatively. However, the S group was associated with worse intraoperative outcomes than the LD group.

## 1. Introduction

Degenerative diseases of the hip joint are more frequently seen due to the prolongation of human life and the increase in the number of patients exposed to musculoskeletal system trauma and diseases. Today, total hip arthroplasty (THA) is the most effective treatment modality for the treatment of advanced hip osteoarthritis due to various reasons [1]. THA techniques include a variety of surgical approaches, including different patient positioning maneuvers, selected with the intent of reducing the soft tissue trauma through shorter incisions [2]. When performing surgical arthroplastic procedures on the hip, the importance of patient positioning cannot be understated [3]. The supine position and the lateral decubitus (LD) position have their associated advantages/disadvantages [4]. However, the superiority of one patient position to another during THA has never been studied in the English literature.

Obesity is an important problem affecting the whole world. Based on data from the 2011–2012 National Health and Nutrition Examination Survey, the age-adjusted prevalence of overweight and obese US adults was 68.5% and 34.9%, respectively [5]. In Turkey, based on the body mass indices (BMIs), the prevalence of obesity has been reported as 29.5% [6]. Obesity presents a challenging problem in orthopedics, as studies have shown that obesity results in a 2-fold increased risk of surgical site infections [7]. Poorer postoperative outcomes have been associated with obesity in a variety of orthopedic surgeries [7]. Although functional status and quality of life are significantly improved after the application of a hip prosthesis, satisfactory results in terms of pain and functional status may not always be obtained in some patients. The reasons for this may include surgical factors or the clinical features of patients. While the majority of the literature has focused on outcomes after THA performed in a direct lateral (DL) approach, there has been a paucity of studies focused on obese patients.

In our study, we aimed to compare the results of supine and lateral decubitus positions for THA performed in a DL approach in patients with BMIs over 25 kg/m^2^ (overweight and obese patients).

## 2. Materials and Methods

### 2.1. Study Design

The study has been conducted in accordance with the principles of the Helsinki Declaration. This research has been approved by the IRB of the authors' affiliated institutions. Written informed consent was obtained from all subjects.

The patients were investigated in two groups according to the surgical position. Overweight or obese patients who had THA using the DL approach in the lateral decubitus position (LD group) (*n* = 54) or in the supine position (S group) (*n* = 45) were retrospectively investigated. The surgeries were performed by two surgeons experienced for more than ten years (OG, SO). The two surgeons who performed the study operated in both the lateral decubitus and supine positions. Surgeon OG performed hip arthroplasty in the LD position in 25, and in the supine position in 29 patients. Surgeon SO performed hip arthroplasty in the LD position in 29, and in the supine position in 16 patients.

Of the 99 patients in both groups, the reasons for an operation were femoral neck fracture in 59, avascular necrosis in 33, and osteoarthritis in 7 patients.

Two experienced senior orthopedic surgeons had previously gained sufficient experience with both studied surgical techniques. The final decision on whether to choose a LD or supine position was made by the treating surgeon according to preference.

#### 2.1.1. Inclusion Criteria

Patients who underwent cementless hip arthroplasty using a DL approach in the lateral decubitus or supine positions, patients with a BMI of more than 25 kg/m^2^, patients undergone unilateral surgery, and patients with no pathology in the contralateral hip were included in the study.

#### 2.1.2. Exclusion Criteria

Patients with Crowe classification 3-4; patients undergoing revision surgery; patients with bilateral hip arthroplasty, inflammatory arthritis, and rheumatic diseases; patients who underwent cement or hybrid hip arthroplasty; patients with inflammatory or infectious diseases such as septic arthritis, rheumatoid arthritis, or ankylosing spondylitis; patients with neurological diseases such as epilepsy; and patients with cardiac pacemakers that may affect the operation were excluded.

### 2.2. Surgical Technique

All patients underwent spinal or combined anesthesia. A proximally porous-coated cementless femoral stem and a press-fit cementless porous-coated acetabular cup fixed with screws were used.

#### 2.2.1. Direct Lateral Approach

Patients to be operated by this method are laid in the *supine* position or *lateral decubitus* position. The skin incision is performed with the hip at 20° flexion, 30° adduction, and 10° internal rotation. The skin incision starts from the proximal 2-3 cm of the greater trochanter and extends for 15-20 cm from the lateral aspect of the femur to its distal part. The length of the skin incision varies depending on the subtrochanteric shortening osteotomy and the obesity status of the patient. The fascia is cut in the direction and length of the skin incision. The capsule is revealed by elevating the anterior 1/3 of the gluteus medius muscle which is attached to the lateral of the trochanter major. The capsule is cut in the direction of the femoral neck, and its superior half is removed. The hip is placed and fixed at 90° flexion and external rotation. The neck is usually cut from 1 cm proximal to the trochanter minor.

### 2.3. Postoperative Period and Rehabilitation

The patients were mobilized with full load-bearing with two Canadian canes on the first postoperative day. The drain was removed at postoperative 24 hours. The patients were treated with enoxaparin sodium with chemical and antiembolic socks, and mechanical venous thromboembolism prophylaxis was applied for 28 days. Controls were performed at six weeks, three months, six months, and 12 months.

### 2.4. Outcome Parameters

Demographic characteristics, age, height, and weight of the patients were recorded; BMIs were calculated. Those whose body mass indices were greater than 30 kg/m^2^ were considered as obese, and those between 25 kg/m^2^ and 30 kg/m^2^ were considered as overweight. Blood loss of patients, amount of transfusion, Harris Hip Scores (preop, 6 weeks, 3 months, 6 months, and 12 months), incision size, surgery time, postoperative acetabular cup inclination angle, femoral stem alignment, follow-up period, hospital stay, preoperative-postoperative leg length inequality, and complication rates (infection, wound site problems, and dislocation rates) were compared.

#### 2.4.1. Harris Hip Score

The patients were evaluated clinically preoperatively and at postoperative 6th week and 3rd, 6th, and 12th months with the Harris Hip Score (HHS). HHS contains questions about pain, function, absence of deformity, and range of motion [8]. Accordingly, the cases were evaluated based on a total score of 100 points regarding pain, function, deformity, and range of motion. In the postoperative period, the results were considered to be excellent (90-100 pts), good (80-89 pts), moderate (70-79 pts), and poor (<70 pts).

### 2.5. Statistical Analysis

Data were analyzed using the MedCalc Statistical Software version 12.7.7 (MedCalc Software bvba, Ostend, Belgium; http://www.medcalc.org; 2013). Parametric tests were applied to data of normal distribution, and nonparametric tests were applied to data of questionably normal distribution. Comparison of two continuous variables with normal distribution was performed by Student's *t*-test. Wilcoxon's test and Mann-Whitney's *U*-test were used to compare two dependent variables. Friedman's test was used to compare groups of dependent continuous variables, and Bonferroni's post hoc analysis was used for multiple comparison tests. Two categorical variables in both groups were compared using the chi-square test. All differences associated with a chance probability of 0.05 or less were considered statistically significant. Data are expressed as mean ± SD or median (interquartile range), as appropriate.

## 3. Results

Ninety-nine consecutive patients met the eligibility criteria for the study. Of the 99 patients (32 males, 67 females) whose charts were reviewed, the mean age was 58.1 ± 12.5 (range 20 to 86) years.

The LD group included 28 right and 26 left hips of 54 patients (18 males, 36 females) with a mean age of 58.2 ± 12.2 (range, 20 to 86) years and with a mean body mass index (BMI) of 29.0 ± 2.1 kg/m^2^. The S group included 24 right and 21 left hips of 45 patients (14 males, 31 females) with a mean age of 57.9 ± 13.0 (range, 23 to 82) years and with a mean BMI of 29.3 ± 2.0 kg/m^2^. Both groups did not differ from each other by means of age, gender, BMI, and affected side (*p* = 0.814, *p* = 0.723, *p* = 0.582, and *p* = 0.833, respectively). Of the 54 patients in the LD group, 36 (66.6%) were overweight, 17 (31.5%) were obese, and 1 (1.9%) was extremely obese. Of the 45 patients in the S group, 29 (64.4%) were overweight and 16 (35.5%) were obese.

The incision length (*p* < 0.001), blood loss (*p* = 0.010), and amount of blood transfused (*p* = 0.002) were significantly higher in the S group than in the LD group. The surgical time was significantly longer in the S group (*p* < 0.001).

None of the patients in the both groups were lost to follow-up.

There were no statistically significant differences between the LD and S groups in terms of pre- and postoperative height, cup inclination, stem alignment, and follow-up period. The median duration of hospital stay was 6.7 (range, 5 to 9) days in the S group and 5.2 (range, 4 to 8) days in the LD group (*p* > 0.05) (Table 1). The change between pre- and postoperative HHS in the LD and S groups was statistically significant (Table 2). Post hoc binary comparison analysis was conducted to investigate the difference between the groups. The values of HHS were significantly increased from the preoperative period to the final follow-up.

For unexpected events or complications in the LD group, superficial infection was observed in one patient, deep vein thrombosis was observed in one patient, and subcutaneous hematoma was observed in two patients. In the S group, superficial infection and subcutaneous hematoma were observed in one patient.

## 4. Discussion

In the present study, we aimed to compare the results of THA applied in the LD and S positions to patients with body mass indices (BMIs) above 25 kg/m^2^. We revealed that one year after THA, comparable functional outcomes were found in two positions, while the S position was associated with worse intraoperative outcomes than the LD position in overweight and obese patients.

Total hip arthroplasty is currently the most outstanding treatment method for hip osteoarthritis, which significantly reduces the comfort of patients due to severe pain and loss of function. In the past years, THA, which was preferred only in elderly patients, has become the primary treatment method in patients with relatively younger and advanced stage osteoarthritis, due to the progression of science and technology, increasing experience, and prolongation of the life expectancy of people [9]. The purpose of THA is to provide the integrity and mechanics of a painless and functional hip joint and to maintain it for the longest period of time.

The supine position and the lateral decubitus position have their associated advantages/disadvantages. However, the superiority of one patient position to another during THA has never been studied in the English literature. Performing surgery in the supine position is an anaesthesiological advantage especially in patients who are in need of extensive monitoring [10]. Also, as the patient is in a supine position, it is easier to orient the native pelvis anatomy, and therefore, theoretically, implantation of the acetabular cup can be done more accurately. As the patient is in a supine position, the use of a c-arm was easier and the image of the pelvis could be taken more accurately compared to when the patient was in a lateral decubitus position. Our result on operation time is consistent with previous studies which reported a longer operation time with the supine position [10, 11]. As the patient is in a supine position, the femur needs to be hyperextended and externally rotated to have access to the canal.

Cup alignment is important for hip stability; inadequate cup alignment increases the possibility of implant dislocation [12]. Studies have revealed that an acetabular cup position differs little from patient to patient in the supine position than in the other patient positions [12, 13]. The acetabular component position is controlled not only by the intraoperative patient positioning but also by the functional pelvic tilt and sagittal plane balance. The supine position for THA facilitates the recreation of the functional pelvic orientation [14]. In the present study, the mean angle of the acetabular cup was 48.1° in patients in the LD group and 47.4° in the S group. This value was reported as 46.4° in the series of Kim et al. [15]. In five cases (2.6%), the angle of the acetabular cup was higher than 55°, and in three of them (1.6%) revision was required. Since in four of these five cases (2.1%), osteoarthritis was found at the setting of subluxation, and in one patient (0.5%) etiology of osteoarthritis was related to developmental hip dislocation, extreme care should be exercised during placement of the acetabular cup.

Femoral stem malposition can lead to an increased risk of dislocation and compression and a decreased range of motion. Femoral anteversion is particularly important in obtaining combined anteversion [16]. Combined anteversion between 25 and 50 degrees should be obtained for the prevention of compression and dislocation [17]. This value was found to be compatible with the literature in both groups. We believe that the surgical position is not effective on femoral stem alignment.

We chose HHS as the primary functional outcome and found no statistical difference between the two positions. In the LD group, the median HHS was 33.7 in the preoperative period and 90.9 in the postoperative follow-up period. The median HHS of the patients in the S group was 32.3 in the preoperative period. In the series of Goldberg et al., Schmalzried and Harris, HHS was 37 in the preoperative evaluation, while good and excellent results had been achieved in 92 and 84.5% of the patients after the average follow-up period of 102 months [18, 19]. Our clinical results were similar to those in the literature.

There are many limitations of our study. First, the number of patients may be insufficient. Secondly, the follow-up time is relatively short. However, the first postoperative year's outcome is the most important in this population due to their high mortality. Lastly, the anteversion of cup and stem was not measured in both groups.

In the present study of THA performed with the DL approach, the LD and S positions had comparable functional outcomes one year postoperatively. However, the S position was associated with worse intraoperative outcomes than the LD position in overweight and obese patients.

## Figures and Tables

**Table 1:** Comparison of study groups for clinical and demographical variables.

	Lateral decubitus group (*n* = 54)	Supine group (*n* = 45)	*p* value
Age (years)	58.2 ± 12.2	57.9 ± 13.0	0.814

Gender (M/F)	18/36	14/31	0.723

Affected side (R/L)	28/26	24/21	0.833

BMI (kg/m^2^)	29.0 ± 2.1	29.3 ± 2.0	0.582

ASA grade	2.02 ± 0.24	1.98 ± 0.38	0.564

Length of incision (cm)	13.9 ± 1.7	19.2 ± 1.5	< 0.001

Surgical time (min)	66.7 ± 15.1	82 ± 15.4	< 0.001

Blood loss (ml)	275.2 ± 66.8	311.6 ± 70.7	0.010

Amount of blood transfused (units)	1.1 ± 0.7	1.6 ± 0.8	0.002

Preop difference in height (cm)	2.2 ± 0.7	2.1 ± 0.8	0.965

Postop difference in height (cm)	0.4 ± 0.5	0.4 ± 0.5	0.966

Acetabular cup inclination angle (°)	48.1 ± 6.5	47.4 ± 5.7	0.555

Stem alignment (°)	1.1 ± 2.0	1.1 ± 2.1	0.997

Follow-up period (months)	35.5 ± 10.0	35.3 ± 9.3	0.838

M: male; F: female; R: right; L: left; BMI: body mass index; ASA: American Society of Anesthesiologists.

**Table 2:** Comparison of study groups for Harris Hip Score for each control visit.

	Lateral decubitus group (*n* = 54)	Supine group (*n* = 45)	*p* value
Preop HHS	33.7 ± 6.7	32.3 ± 5.5	0.424

Postop 6-week HHS	61.8 ± 7.5	61.0 ± 4.9	0.443

Postop 3-month HHS	78 ± 3.8	78.4 ± 3.7	0.574

Postop 6-month HHS	85.7 ± 4.8	86.2 ± 4.9	0.397

Postop 12-month HHS	90.9 ± 3.4	90.6 ± 4.4	0.666

*p* value	<0.001	<0.001	

HHS: Harris Hip Score.

## Data Availability

The data used to support the findings of this study are available from the corresponding author upon request.
